# Neuromelanin, aging, and neuronal vulnerability in Parkinson's disease

**DOI:** 10.1002/mds.27776

**Published:** 2019-06-28

**Authors:** Miquel Vila

**Affiliations:** ^1^ Neurodegenerative Diseases Research Group Vall d'Hebron Research Institute–Center for Networked Biomedical Research on Neurodegenerative Diseases Barcelona Spain; ^2^ Department of Biochemistry and Molecular Biology Autonomous University of Barcelona Barcelona Spain; ^3^ Catalan Institution for Research and Advanced Studies Barcelona Spain

## Abstract

Neuromelanin, a dark brown intracellular pigment, has long been associated with Parkinson's disease (PD). In PD, neuromelanin‐containing neurons preferentially degenerate, tell‐tale neuropathological inclusions form in close association with this pigment, and neuroinflammation is restricted to neuromelanin‐containing areas. In humans, neuromelanin accumulates with age, which in turn is the main risk factor for PD. The potential contribution of neuromelanin to PD pathogenesis remains unknown because, in contrast to humans, common laboratory animals lack neuromelanin. The recent introduction of a rodent model exhibiting an age‐dependent production of human‐like neuromelanin has allowed, for the first time, for the consequences of progressive neuromelanin accumulation—up to levels reached in elderly human brains—to be assessed in vivo. In these animals, intracellular neuromelanin accumulation above a specific threshold compromises neuronal function and triggers a PD‐like pathology. As neuromelanin levels reach this threshold in PD patients and presymptomatic PD patients, the modulation of neuromelanin accumulation could provide a therapeutic benefit for PD patients and delay brain aging. © 2019 The Author. *Movement Disorders* published by Wiley Periodicals, Inc. on behalf of International Parkinson and Movement Disorder Society.

## Neuromelanin and PD: A 100‐Year‐Old Relationship

A century ago, in 1919, Konstantin Tretiakoff reported for the first time in his remarkable doctorate thesis the presence of a marked loss of pigmented neurons in the substantia nigra (SN), visible with the naked eye, in the brains of Parkinson's disease (PD) patients.^1^ Although this finding failed to gain him any significant recognition during his lifetime, his observation remains to this day the cardinal pathologic diagnostic criterion for PD.^1^ The loss of nigral pigmented neurons, which we now know produce the neurotransmitter dopamine, leads to the classical motor symptoms of PD and constitutes the only robust clinico‐pathological correlation associated with the disease.^1^ The pigment contained within these neurons, termed *neuromelanin* because of its similar appearance to cutaneous melanin, is so abundant in the SN of the human brain that this structure can be seen macroscopically as a darkened area (hence the origin of the name given to this brain region).^2^ Later, other neuromelanin‐containing neurons in different brain regions were also found to consistently degenerate in PD; these include the noradrenergic neurons of the locus coeruleus and dorsal motor nucleus of the vagus, leading to characteristic nonmotor symptoms of the disease.^3^, ^4^ In contrast, neuronal loss in nonmelanized brain regions appears either inconsistent, not specific to PD, or secondary to the loss of interconnected neuromelanin‐containing neurons.^5^, ^6^, ^7^

In the normal human midbrain, dopamine‐producing cell groups markedly differ from each other in terms of the percentage of neuromelanin‐pigmented neurons they contain.^8^, ^9^, ^10^ In PD, the estimated cell loss in these groups correlates directly with the percentage of neuromelanin‐pigmented neurons normally present in them.^8^, ^9^, ^10^ Likewise, within each neuromelanin‐containing cell group, there is greater relative sparing of weakly pigmented than of strongly melanized neurons.^8^ For instance, the loss of catecholaminergic neurons in PD is severe in the SN pars compacta (SNpc), in which virtually all neurons are pigmented, and almost undetectable in the central gray substance, in which most catecholaminergic neurons are not pigmented.^9^ In the peri‐ and retro‐rubral regions of the midbrain, which contain pigmented and nonpigmented catecholaminergic neurons in similar proportions, the population of melanized catecholaminergic neurons is significantly decreased, whereas the total population of catecholaminergic neurons devoid of neuromelanin is not affected.^9^ Similarly, dopaminergic neurons in the ventral tegmental area, which are largely spared in PD, produce minimal neuromelanin over a lifetime.^8^, ^11^

Classical Lewy bodies (LB), that is, α‐synuclein‐containing intracytoplasmic inclusions that constitute the pathological hallmark of PD besides cell loss, and their presumed precursor structures, termed *pale bodies*, typically appear within the intracellular areas of the cytoplasm in which neuromelanin accumulates and form in close physical association with this pigment.^12^ Consistent with these observations, studies on human brains have shown that α‐synuclein redistributes to the neuromelanin pigment in early stages of PD and becomes entrapped within neuromelanin granules.^13^, ^14^, ^15^ Further linking the PD neuropathology with neuromelanin, neuroinflammatory changes occurring in PD brains, such as activation of innate (ie, microgliosis) and adaptive (ie, lymphocyte infiltration) immune responses, are highly localized within neuromelanin‐containing areas and only sparingly observed in nonmelanized regions.^16^ For instance, extracellular neuromelanin released from dying neurons activates microglia in PD brains to phagocytize and degrade/eliminate this pigment.^17^ Also, neuromelanin‐containing SNpc neurons from PD brains exhibit Immunoglobulin G accumulation and antigenic major histocompatibility complex class I expression in a manner directly correlated to neuronal loss.^18^ In addition, antimelanin antibodies are increased in the sera of PD patients.^19^

The PD pathogenesis thus appears to be inextricably linked to the presence of neuromelanin. However, despite the close and long‐established association between neuromelanin and PD, the physiological significance of neuromelanin and its potential contribution to PD pathogenesis remain unknown because, in contrast to humans, laboratory animal species commonly used in experimental research, such as rodents, lack neuromelanin. Although neuromelanin is actually present in some other species as varied as monkeys,^20^, ^21^ dolphins,^22^ and frogs,^23^ the highly abundant quantity of neuromelanin in the brain stem seems unique to humans, as a dark pigmentation of this brain area is not apparently observed in other animal species at the macroscopic level.^20^ In fact, in the mammalian brain, neuromelanin accumulation increases progressively as the evolutionary relation to man becomes closer,^20^ and humans are the only species that naturally develops PD.^24^ Consequently, a factor so intimately linked to PD such as neuromelanin has been surprisingly neglected to date in experimental in vivo paradigms of the disease.

## Synthesis of Neuromelanin: Mechanisms and Significance

In contrast to the widespread distribution of other brain pigments such as lipofuscin, neuromelanin is restricted to catecholamine‐producing regions of the brain and forms only in neurons. Neuromelanin first becomes observable in the human SNpc at approximately 3 years of age and progressively accumulates over time within the cells in which it has been produced, as neurons apparently lack the mechanisms for degrading or eliminating this pigment.^25^, ^26^ As a consequence, intracellular neuromelanin builds up with age until occupying most of the neuronal cytoplasm.^25^ Although aging is the main risk factor for developing PD,^27^ the molecular substrate linking PD with aging is currently unknown.

Melanins (from the Greek word *melanos* [“black”]) are a group of complex, brown‐black pigmented biopolymers that include eumelanin (the pigment associated with dark hair and skin), pheomelanin (characteristic of red and blond hair), and neuromelanin (ie, brain melanin, which is thought to contain a pheomelanin core and eumelanin surface^28^). Melanins derive from a complex biosynthetic pathway initiated with the hydroxylation of L‐tyrosine to L‐dihydroxyphenylalanine (l‐dopa). After this common step, l‐dopa serves as a precursor to both melanins and catecholamines, acting along separate pathways. Although the synthesis of peripheral melanin pigments (eg, in skin and hair) is relatively well understood, the mechanism leading to neuromelanogenesis is still a matter of speculation. Both neuromelanin and peripheral melanins are produced as oxidative products downstream from l‐dopa. In fact, neuromelanin synthesis is regarded as a protective antioxidant mechanism to remove potentially toxic oxidized dopamine species, such as quinones and semiquinones, by their conversion into neuromelanin.^29^ However, although peripheral melanogenesis occurring within specialized cells (ie, melanocytes) is known to result from an enzymatically driven biosynthetic pathway in which tyrosinase is the key, rate‐limiting enzyme,^30^ it is widely assumed that neuromelanin is instead produced by spontaneous nonenzymatic dopamine auto‐oxidation.^31^ Several observations argue, however, against the latter concept. For instance, the distribution of neuromelanin does not fully match that of the dopamine‐producing enzyme tyrosine hydroxylase (TH), with many dopamine and noradrenergic neurons completely lacking neuromelanin.^8^, ^32^, ^33^, ^34^ In addition, neuromelanin is not observed in most animal species despite the presence of dopamine and other catecholamines in these animals.^20^ Although this could be related to differences in lifespan or rate of catecholamine synthesis between species, experimentally induced increases in dopamine and/or oxidized dopamine in mice and rats, achieved either with chronic l‐dopa treatment^35^, ^36^ or by genetically enhancing TH activity,^37^ are not sufficient by themselves to produce neuromelanin in these animals, as might be expected if neuromelanin represents a mere process of auto‐oxidized dopamine. Along this line, it is worth noting that George C. Cotzias's initial use of l‐dopa in PD patients was apparently aimed at restoring neuromelanin levels, and not dopamine, as he thought that PD might result from the loss of the neuromelanin pigment in the SNpc.^38^ This approach, however, failed to reinstate the missing pigment in PD brains (but it provided instead a dramatic antiparkinsonian effect by replacing the depleted dopamine, thus establishing a revolutionary new treatment for PD, although apparently for the wrong reason).^38^

The identification of specific phases and changes in the rate of neuromelanin production over time in humans^25^, ^26^ suggests the regulation of neuromelanin production and turnover, possibly through enzymatic processes,^26^ as is the case for all other melanin pigments.^39^ Interestingly, tyrosinase expression might not be restricted to melanocytes but could also be present, although at very low levels, in the brain, including the human SNpc.^40^, ^41^, ^42^, ^43^ For instance, brain tyrosinase messenger ribonucleic acid (mRNA) expression has been reported in human SN by reverse transcription polymerase chain reaction/real‐time polymerase chain reaction, although at barely detectable levels.^41^, ^42^, ^43^ Similarly, 3 different microarray experiments in human brain tissue (Entrez_id: 7299; Allen Brain Institute Seattle, Washington), nonhuman primate brain tissue (Entrez_id: 705792; National Institutes of Health [NIH] Blueprint Non‐human Primate [NHP] Atlas), and developing human prenatal tissue (Entrez_id: 7299; BrainSpan Atlas of the developing human brain) detected the presence of some tyrosinase transcripts in the brain. Consistent with the very low levels at which tyrosinase mRNA might be expressed, if at all, in the brain, the actual protein has not been detected in the human brain when using immunohistochemistry or Western blot.^44^, ^45^ Similarly, mass spectrometry techniques failed to detect significant amounts of typical enzymes and proteins involved in melanogenesis, including tyrosinase, in isolated neuromelanin‐containing organelles from human SN.^15^, ^46^, ^47^ In contrast, tyrosinase enzymatic activity has been reported in brain extracts from human SN,^41^ although at 100,000 times lower than in melanocytes.^48^ These inconsistent results could be potentially attributed to the very low levels at which tyrosinase might be actually expressed, if at all, in the brain and further experiments are thus warranted to unambiguously demonstrate or refute the existence of tyrosinase in the human brain. Although it is currently unknown whether tyrosinase may contribute to neuromelanin synthesis, this possibility is theoretically conceivable because tyrosinase not only mediates the hydroxylation of tyrosine and the oxidation of l‐dopa necessary for the formation of peripheral melanins but also is able to oxidize dopamine's catechol ring, which is an essential event required for neuromelanin synthesis.^49^ In addition, a rare loss‐of‐function mutation in tyrosinase linked to albinism has been recently associated with an increased risk for PD, which has been attributed to a possible inability of this tyrosinase variant to synthesize neuromelanin in these patients and the subsequent accumulation of potentially toxic dopamine‐derived species that cannot be detoxified by conversion into neuromelanin.^50^ On the other hand, arguing against a potential role of tyrosinase in neuromelanin synthesis, the presence of neuromelanin was reported in the brains of 2 subjects with albinism,^51^ which are usually assumed to lack tyrosinase activity. However, that report was published in the pregenetics diagnostic era and, in absence of specific information about the genetic type of albinism suffered by these cases, it cannot be excluded that residual tyrosinase activity was actually present in these subjects.^52^ Indeed, because of the clinical overlap between oculocutaneous albinism (OCA) subtypes, molecular diagnosis is necessary to establish the specific gene defect and thus the OCA subtype. Among the most common types of albinism, only the OCA1 subtype is actually caused by mutations in the tyrosinase gene.^53^ In turn, within OCA1 subjects, tyrosinase activity is completely abolished only in the OCA1A subtype while in OCA1B some enzymatic activity still remains, allowing some accumulation of melanin pigment over time.^53^ In this context, at least one of the subjects examined by Foley and Baxter was diagnosed with “partial albinism,”^51^ implying that some residual tyrosinase activity could have been present in these brains. Therefore, whether tyrosinase or other related enzymes may contribute to neuromelanin synthesis remains an open question.

## Modeling Neuromelanin Production In Vivo: Age‐Dependent Intracellular Neuromelanin Accumulation Drives PD Pathology

**Figure 1:**
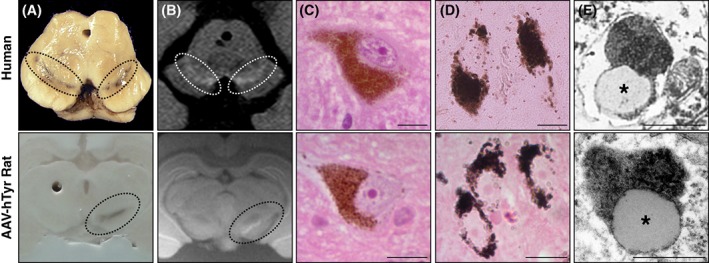
Human‐like neuromelanin production in tyrosinase‐overexpressing rats. Representative images from elderly human control brains (top) and brains from rats unilaterally injected with an adeno‐associated viral (AAV) vector expressing human tyrosinase (hTyr) above the right substantia nigra pars compacta (SNpc; bottom). Humans and AAV‐hTyr‐injected rats exhibit analogous neuromelanin production from a macroscopic (**A,B**), microscopic (**C,D**), and ultrastructural (**E**) point of view. No neuromelanin is observed in the contralateral (noninjected) hemisphere of AAV‐hTyr‐injected rats, as rodents normally lack this pigment. (**A**) Unstained midbrain (dashed outline, melanized SNpc). (B) Neuromelanin‐sensitive high‐resolution T1‐weighted magnetic resonance imaging (dashed outline, melanized SNpc visualized as an hyperintense area). (**C**) Hematoxylin‐eosin stained 5‐μm‐thick SNpc sections (unstained neuromelanin is seen in brown). (**D**) Masson‐Fontana melanin staining in 5‐μm‐thick SNpc sections (neuromelanin is seen in dark brown). (**E**) Electron microscopy (neuromelanin, electron dense matrix; asterisks, associated lipid droplets). Scale bars, 12.5 μm (**C,D**), 500 nm (**E**). Adapted from ref. 43.

**Figure 2:**
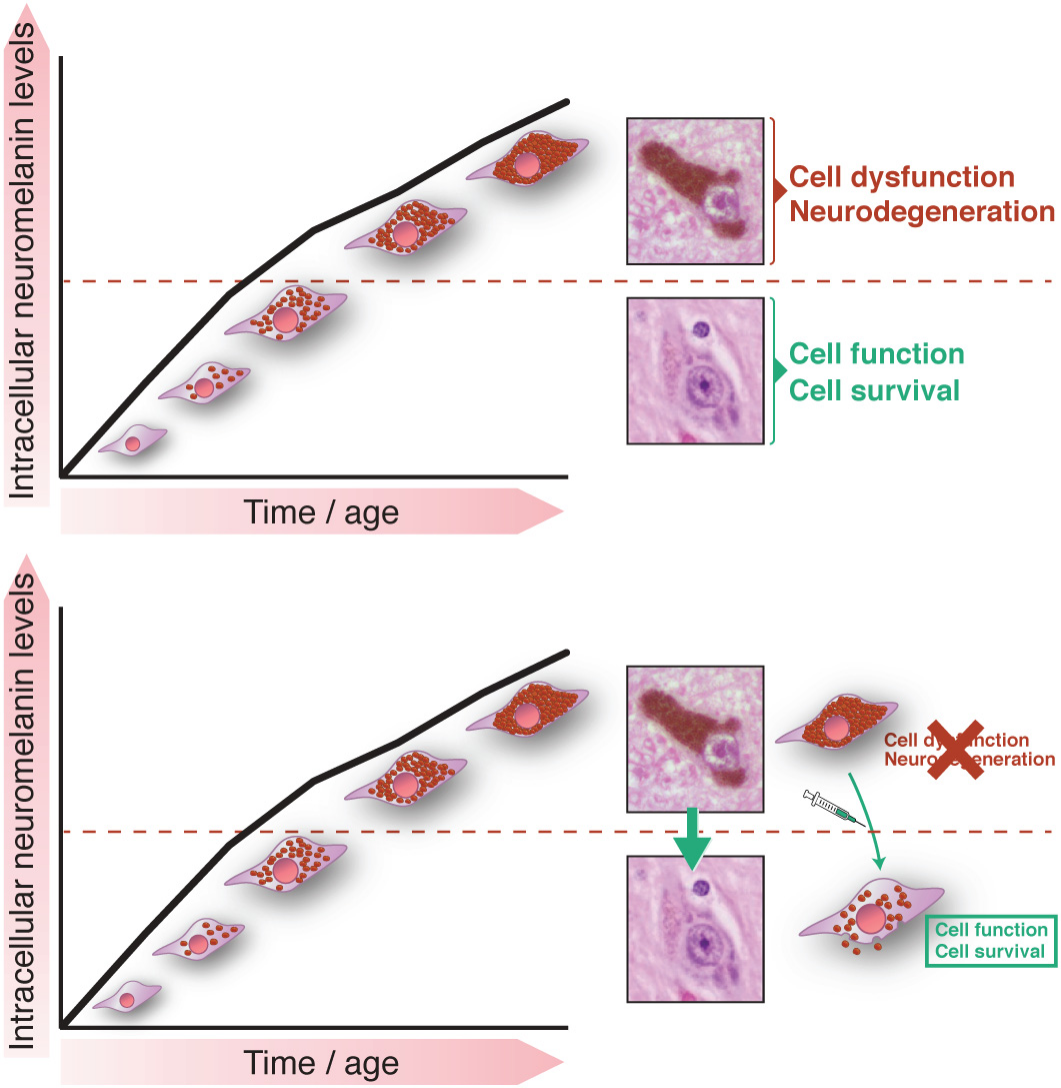
Putative pathogenic threshold of intracellular neuromelanin accumulation. (Top) Age‐dependent intracellular neuromelanin accumulation in human tyrosinase (hTyr)–overexpressing rodents is associated with Parkinson's disease–like neuronal dysfunction and degeneration when reaching a specific threshold of accumulation (dotted red line)^43^. In aged postmortem brains from healthy individuals intracellular neuromelanin levels are maintained below this pathogenic threshold while in age‐matched brains from Parkinson's disease patients and pre–Parkinson's disease (ie, incidental Lewy Body disease) subjects intracellular neuromelanin levels appear above this pathogenic threshold.^43^ Please note that such a pathogenic threshold would be valid regardless of the actual mechanism of neuromelanin synthesis (whether enzymatic or nonenzymatic). (Bottom) Neuronal “rejuvenation” strategies aimed at reducing intracellular levels of neuromelanin back to younger states (ie, below the pathogenic threshold of neuromelanin accumulation) should prevent, halt, or delay neuronal dysfunction and degeneration linked to both Parkinson's disease and brain aging. Because of the putative protective role of neuromelanin synthesis (ie, to remove potentially toxic dopamine‐derived oxidative species from the cytosol), such strategies should probably favor the elimination of neuromelanin once it has already been produced (for instance, by promoting the exocytosis of neuromelanin‐filled lysosomal/autophagic structures)^43^ instead of inhibiting neuromelanin synthesis per se. Photomicrographs correspond to hematoxylin‐eosin stained human brain sections illustrating dopaminergic nigral neurons with high or low levels of intracellular neuromelanin.

**Figure 3:**
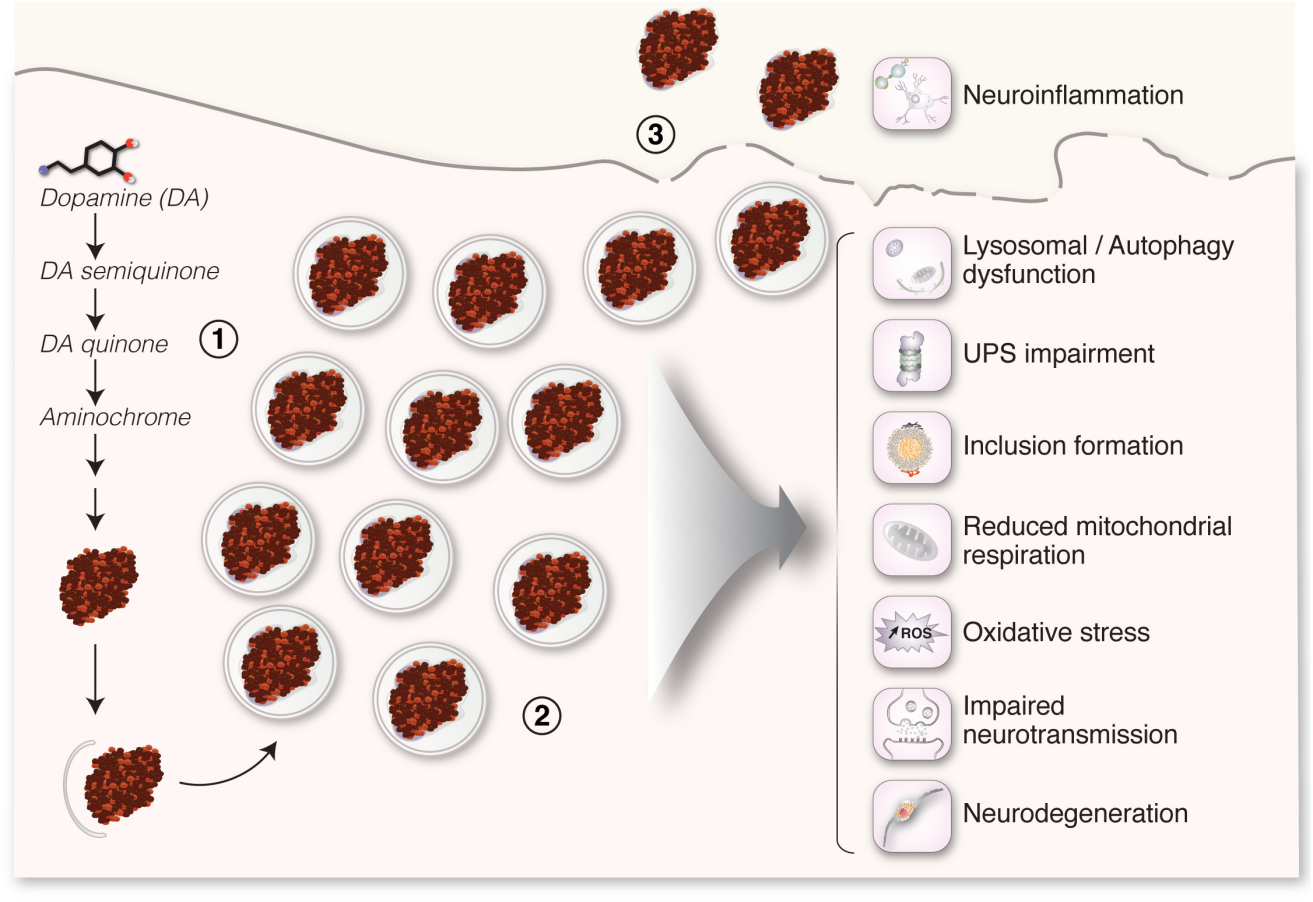
Molecular mechanisms of neuromelanin‐linked neurotoxicity. (1) Dopamine oxidation generates many o‐quinones, which can be potentially toxic to neurons. However, the continuous conversion of these oxidized dopamine species into neuromelanin prevents their pathological accumulation in the cytoplasm,^43^ which is consistent with a protective antioxidant role of neuromelanin synthesis. (2) The continuous intracellular build‐up of neuromelanin within undegraded lysosomal‐autophagic structures, until occupying most of the neuronal cytoplasm, may physically interfere with intracellular trafficking/communication and ultimately exhaust the vesicular storage capacity of the cell, leading to a general failure of cellular proteostasis coursing with lysosomal/autophagy dysfunction, ubiquitin‐proteasome (UPS) impairment, intracellular inclusion body formation, reduced mitochondrial respiration, increased production of reactive oxygen species, impaired neurotransmission, and neurodegeneration. All of these pathological changes are observed in neuromelanin‐producing human tyrosinase–overexpressing animals and Parkinson's disease patients.^43^ (3) Extracellular neuromelanin released from dying neurons activates microglia that phagocytize this pigment^43^ (ie, neuronophagia) and may trigger an antigenic response able to induce a selective T cell‐mediated cytotoxic attack against neuromelanin‐laden neurons,^18^ potentially contributing to the progression of the neurodegenerative process.

To assess the potential role of neuromelanin in PD pathogenesis, it would first be necessary to develop an in vivo model of human‐like neuromelanin production, up to the abundant levels reached in elder humans, as no such animal model currently exists, experimentally or naturally. Considering the failure by previous studies to produce neuromelanin in vivo solely by increasing the levels of dopamine and oxidized dopamine,^35^, ^36^, ^37^ we recently opted as a potential alternative strategy for overexpressing human tyrosinase (hTyr) via a viral vector in the SNpc of both rats and mice, which normally lack neuromelanin.^43^ Strikingly, hTyr‐overexpressing rodents exhibited an age‐dependent production and accumulation of a neuromelanin‐like pigment within nigral dopaminergic neurons that was virtually analogous to human neuromelanin^43^ (henceforth referred to as neuromelanin; see Fig. 1). Similar to humans, neuromelanin from hTyr‐overexpressing rodents (1) could be visualized with the naked eye as a darkened SNpc area^43^ (Fig. 1A); (2) was detected as an hyperintense area by neuromelanin‐sensitive high‐resolution T1‐weighted magnetic resonance imaging (MRI)^43^ (Fig. 1B); (3) accumulated progressively over time within SNpc dopaminergic neurons until occupying most of their neuronal cytoplasm, reaching levels consistent with those seen in elderly humans^43^ (Fig. 1C); (4) stained prominently with the melanin marker Masson‐Fontana, which reflects the ability of neuromelanin to chelate metals^43^ (Fig. 1D); and (5) was encapsulated within autophagic structures exhibiting, at the ultrastructural level, an electron‐dense matrix associated with characteristic lipid droplets^43^ (Fig. 1E). The striking resemblance between human neuromelanin and neuromelanin from hTyr‐overexpressing rodents, combined with the fact that hTyr overexpression is sufficient in itself to produce neuromelanin in these animals (as opposed to the lack of neuromelanin by solely increasing dopamine/oxidized dopamine levels), supports the possibility that brain tyrosinase or other related enzymes might potentially contribute to neuromelanin formation in humans. Whatever the case may be, these animals represent the first experimental in vivo model of the age‐dependent production and accumulation of human‐like neuromelanin within PD‐vulnerable nigral neurons at levels up to those reached in elderly humans. The model thus constitutes a unique experimental tool enabling assessment of the possible consequences of neuromelanin production/accumulation on neuronal function and viability.

In this context, one of the main questions to be addressed concerns how a neuron containing very high levels of intracellular neuromelanin, to the point where the neuromelanin occupies most of the neuron's cytoplasm, can function properly. The answer is, in fact, that it cannot. Indeed, it was found in hTyr‐overexpressing rodents that the progressive intracellular build‐up of neuromelanin ultimately compromised neuronal function when allowed to accumulate above a specific threshold, eventually triggering in an age‐dependent manner the main pathological features of PD, including hypokinesia, LB‐like inclusion formation and nigrostriatal neurodegeneration.^43^ By ~4 months, nigrostriatal denervation in hTyr‐overexpressing rodents was equivalent to that seen in early PD patients (<10 years’ evolution), and by ~2 years the extent of denervation reached levels comparable to those seen in advanced PD (>20 years’ evolution).^43^, ^54^ In parallel to dopaminergic cell death, these animals exhibited other neuropathological features typical of aged and PD human brains, including (1) extracellular neuromelanin, released from dying neurons^43^; (2) neuronophagia (ie, extracellular neuromelanin surrounded by, or within, activated microglia), indicative of an active, ongoing neurodegenerative process^43^; and (3) perivascular neuromelanin, resulting from the migration of neuromelanin‐filled microglia to blood vessels to remove extracellular neuromelanin from the brain.^43^

Prior to degeneration, neuromelanin‐filled neurons from hTyr‐overexpressing rodents exhibited early signs of neuronal dysfunction similar to those occurring in PD patients, including impaired dopamine release, axonal swelling, decreased striatal levels of dopamine transporter, and phenotypic loss of TH expression, all of which was accompanied by early motor deficits in these animals.^43^ The occurrence of neuronal dysfunction before overt cell death may have important therapeutic implications, as it provides a therapeutic window in which neuronal function could be potentially restored before the actual loss of the cell. Interestingly, PD‐type inclusion body formation in hTyr‐overexpressing rodents, comprising both pale bodies–like and LB‐like structures exclusively observed within neuromelanin‐filled neurons, peaked at the time of early neuronal dysfunction and was substantially reduced once neurodegeneration was established.^43^ This observation suggest that inclusion‐containing neurons are those that become dysfunctional and preferentially degenerate in these animals. Consistent with this, inclusion‐containing neurons in both humans^55^ and hTyr‐overexpressing rodents^43^ often exhibit TH downregulation, which reflects neuronal dysfunction at early stages of neurodegeneration.^8^ In addition, similar to hTyr‐overexpressing rodents, the number of neuronal inclusions in PD brains at advanced stages of the disease is much lower than that observed in cases involving early PD.^16^

Overall, the unprecedented use of neuromelanin‐producing hTyr‐overexpressing rodents revealed that age‐dependent neuromelanin production within SNpc dopaminergic neurons is associated with neuronal dysfunction and progressive nigrostriatal neurodegeneration, equivalent to that occurring in PD patients, once a certain threshold of intracellular neuromelanin accumulation is reached (Fig. 2). Relevant to humans, intracellular neuromelanin levels reach this threshold in both PD patients and subjects with incidental LB disease (ie, clinically healthy individuals exhibiting LB pathology at autopsy who are considered to represent early, presymptomatic stages of PD).^43^ In contrast, in healthy elderly individuals, intracellular neuromelanin levels remain below this threshold.^43^ Although previous studies reported a decrease in neuromelanin levels in dopaminergic neurons from PD patients,^10^ this was observed only in those neurons in the final stages of degeneration.^13^ In contrast, in adjacent, vulnerable (but yet morphologically intact) dopaminergic neurons, the neuromelanin density was augmented,^13^ further supporting the notion that increased intracellular neuromelanin levels may precede dopaminergic cell death and predispose these neurons to degeneration in PD. Please note that in these studies intracellular neuromelanin levels were measured by optical densitometry within individual neuromelanin‐containing neurons,^10^, ^13^, ^43^ as opposed to regional quantifications of total SNpc neuromelanin,^56^ the latter being dramatically decreased in PD brains because of the massive loss of neuromelanin‐containing dopaminergic neurons.^56^ Supporting a pathogenic role for intracellular neuromelanin accumulation when allowed to accumulate above a specific threshold within individual neurons, the lowering of intracellular neuromelanin to levels below this pathogenic threshold in hTyr‐overexpressing rodents, in part by promoting the release of intracellular neuromelanin to the outside of the cell (see the next section for details), diminished PD‐linked inclusion formation, attenuated nigrostriatal neurodegeneration, and reversed hypokinesia in these animals.^43^ These results demonstrate the feasibility and therapeutic potential of modulating intracellular neuromelanin levels in vivo and indicate that strategies to maintain or decrease intracellular neuromelanin to levels below its pathogenic threshold may provide unprecedented therapeutic opportunities to prevent, halt, or delay neuronal dysfunction and degeneration linked to PD and brain aging (Fig. 2).

## Mechanisms of Neuromelanin‐Linked Neurodegeneration

The oxidation of dopamine to form neuromelanin generates many o‐quinones, which can be potentially toxic to neurons^31^ (Fig. 3). Indeed, dopamine oxidation has long been proposed as a leading pathogenic factor in PD (for a review on this specific topic, see, for instance, ref. 31). Therefore, the pathological PD phenotype observed in neuromelanin‐producing hTyr‐overexpressing rodents could potentially result from the production of toxic dopamine‐derived intermediate species by overexpressed hTyr, with neuromelanin accumulation in these animals simply representing an inert by‐product of these reactions. However, hTyr‐overexpressing rodents showed no significant accumulation of oxidized dopamine species, either preceding or concomitant with neuronal dysfunction/degeneration.^43^ In fact, the significant accumulation of such potentially toxic species in these animals was actually prevented by their continuous conversion into neuromelanin,^43^ this being consistent with a putative protective antioxidant role of neuromelanin synthesis.^31^ Further arguing against a major pathogenic contribution of dopamine‐mediated toxicity in hTyr‐overexpressing rodents, it has been recently reported by 2 independent groups that dopamine‐induced toxicity in rodents, either by chronic l‐dopa treatment^35^ or by enhancing TH activity,^37^ is only observed in animals displaying additional PD‐related alterations, such as DJ‐1 deficiency^35^ or overexpression of PD‐linked A53T mutant α‐synuclein,^37^ but not in regular wild‐type animals^35^, ^37^ (as opposed to the PD phenotype observed in wild‐type rodents overexpressing hTyr^43^). Indeed, although high concentrations of dopamine can be acutely toxic, mostly in vitro*,* 11 the chronic enhancement of dopamine levels does not cause dopaminergic nerve terminal or cell body loss in vivo*,* 35, 36, 37 probably because of compensatory protective mechanisms.^57^ In addition, dopamine‐induced toxicity has been shown to be dependent on α‐synuclein expression,^11^, ^35^, ^37^, ^57^ in contrast to the nonessential role of α‐synuclein for neuromelanin‐linked pathology in hTyr‐overexpressing animals^43^ (see later for details). Although a potential pathogenic contribution of oxidized dopamine species in neuromelanin‐producing hTyr‐overexpressing rodents cannot be completely ruled out, because neuromelanin production is indissociable from dopamine oxidation, other explanations for the observed pathogenicity in these animals could be envisaged.

In both humans and hTyr‐overexpressing rodents, intracellular neuromelanin accumulates within lysosomal‐autophagic structures in an attempt by the cell to degrade this pigment^15^, ^58^ (Fig. 3). However, because of its insoluble nature, neuromelanin cannot be degraded by lysosomes and remains trapped within undegraded or partly degraded autophagic structures that accumulate with age.^58^ The continuous build‐up of neuromelanin within autophagic compartments may ultimately exhaust the vesicular storage capacity of the cell, interfere with lysosomal proteases and other degradative pathways, impair intracellular vesicular trafficking, and alter endocytic/secretory tasks.^58^, ^59^ Supporting this concept, l‐dopa–induced toxicity in vitro is only observed at doses associated with neuromelanin formation^11^ and has been attributed to an interference by neuromelanin with intracellular neurotrophin signaling, and not to an acute toxic effect of l‐dopa per se.^59^ Neuromelanin could thus be toxic to aminergic neurons insofar as it physically interferes with intracellular communication,^60^, ^61^ causing a “macromolecular crowding” effect,^62^ thereby interfering with the synthesis and degradation of cellular proteins.^63^ Consistent with this concept, the progressive accumulation of neuromelanin‐filled autophagic structures in hTyr‐overexpressing cells was associated with parallel decreases in both autophagic and ubiquitin‐proteasome (UPS) degradation system activities, leading to a general failure of cellular proteostasis and subsequent inclusion formation.^43^ Relevant to PD, both autophagy and UPS systems are impaired in neuromelanin‐laden, but not in nonmelanized, regions from postmortem PD brains.^64^, ^65^ Impairment of autophagy and UPS is indicative of a general, late‐stage proteostasis failure in which cell function and survival become compromised.^66^ For instance, mitochondrial quality control is tightly linked to proteolytic cytosolic systems; if the latter are blocked, damaged mitochondria gradually accumulate.^66^ Consistent with this, neuromelanin‐producing cells also exhibit impaired mitochondrial respiration and increased production of reactive oxygen species, 2 pathogenic events that have been consistently linked to PD.^43^ Confirming a pivotal pathogenic role for impaired proteostasis in neuromelanin‐laden cells, enhancement of lysosomal‐mediated proteolysis in hTyr‐overexpressing rodents by overexpression of transcription factor EB (TFEB) reduced intracellular neuromelanin to levels below the pathogenic threshold, attenuated PD‐like inclusion formation, prevented nigrostriatal neurodegeneration, and reversed motor impairment in these animals.^43^ TFEB is a master regulator of autophagy that induces the biogenesis of lysosomes and autophagosomes, boosts autophagic cellular clearance, and modulates general proteostasis^67^, ^68^, ^69^, ^70^ as part of a pleiotropic range of effects.^71^ Interestingly, some of TFEB's effects at promoting cellular clearance involve the activation of lysosomal exocytosis, a process in which lysosomes fuse with the plasma membrane and empty their contents to the outside of the cell.^69^, ^72^ Because neuromelanin is trapped within lysosomal‐autophagic structures, TFEB‐induced reduction of intracellular neuromelanin levels occurred, at least in part, by the exocytosis of neuromelanin‐filled lysosomes outside the cell.^43^ This approach may thus represent a potential therapeutic tool to reduce intracellular neuromelanin levels below their pathogenic threshold of accumulation without interfering with neuromelanin synthesis.

## α‐Synuclein in PD: A Key Factor or a Misleading Clue?

Given the formation of α‐synuclein‐positive inclusions and oligomers in neuromelanin‐producing hTyr‐overexpressing rodents and the pathogenic role attributed to α‐synuclein aggregation in PD, it is feasible that the pathological phenotype of these animals could be potentially mediated by α‐synuclein. Strikingly, α‐synuclein was found to be dispensable for PD‐like inclusion formation and nigrostriatal degeneration, as well as for neuromelanin production, in hTyr‐overexpressing rodents.^43^ Indeed, the induction of neuromelanin production by hTyr overexpression in α‐synuclein‐deficient mice resulted in levels of pale bodies/LB‐like inclusion formation and nigrostriatal degeneration comparable to those seen in hTyr‐overexpressing wild‐type animals.^43^ However, although Lewy pathology in the latter animals displayed LB markers such as p62, ubiquitin, or α‐synuclein, in hTyr‐overexpressing α‐synuclein‐deficient mice Lewy pathology displayed p62 and ubiquitin but lacked α‐synuclein.^43^ These results indicate that α‐synuclein, which is currently regarded as a major component of LB, might not in fact be required for LB formation or for neurodegeneration (whether in the absence of α‐synuclein these structures can still be called “LB” is a matter of semantics). Consistent with a dispensable role of α‐synuclein in the pathogenesis of PD, α‐synuclein accumulation correlates poorly with clinical symptoms and synaptic/neuronal loss in PD patients.^73^ Also, although α‐synuclein‐derived peptides have been proposed to act as antigenic epitopes capable of inducing a selective T‐cell‐mediated cytotoxic attack in PD patients,^74^ the activation of both innate and adaptive immune responses in postmortem PD brains is localized within neuromelanin‐containing areas and absent from nonmelanized regions such as the cortex despite the latter exhibiting abundant α‐synuclein depositions.^16^, ^18^ Furthermore, α‐synuclein pathology is not mandatory for the development of PD, as shown by some genetic PD cases caused by Leucine‐rich repeat kinase 2 and parkin mutations that course with pure nigrostriatal degeneration without Lewy pathology.^75^ Moreover, phosphorylation of α‐synuclein, which is widely considered to be an index of PD pathology, (1) can also be seen in tissue from control subjects^76^; (2) may result from a nonspecific cross‐reaction with other phospho‐proteins, such as phosphorylated neurofilaments^77^; and (3) does not affect neuronal survival in in vitro PD models.^78^ In addition, peripheral α‐synuclein aggregates from PD patients, which are believed to spread to the brain and initiate PD, lack the capacity to promote α‐synuclein pathology, propagate between neuronal networks, or induce neurodegeneration when inoculated into experimental animals.^79^ Finally, in the original studies reporting an apparent transmission of LB pathology from host PD patients to embryonic mesencephalic neurons grafted into the striatum many years earlier,^80^, ^81^ which gave rise to the currently prevalent pathogenic hypothesis of cell‐to‐cell transmission of α‐synuclein, LB formation occurred exclusively within grafted neurons that contained adult levels of neuromelanin and was not observed in nonmelanized grafted cells or intrinsic striatal neurons of the host. Therefore, the latter studies may not in fact reflect a host‐to‐graft propagation of α‐synuclein as was widely interpreted but, rather, indicate that grafted cells had reached pathogenic levels of intracellular neuromelanin accumulation. Overall, it is possible that α‐synuclein pathology in PD may merely represent an epiphenomenon within a more general PD‐causing pathogenic process, such as the one linked to neuromelanin accumulation.^43^ Although this concept does not undermine the value of α‐synuclein as a potential biomarker of PD, it raises the importance of reassessing the value of α‐synuclein as a potential therapeutic target for the disease.

## Implications of Neuromelanin‐Driven Pathology for PD and Brain Aging

The recent development of a rodent model producing human‐like neuromelanin has allowed for the consequences of age‐dependent neuromelanin accumulation up to levels reached in elderly humans to be experimentally assessed for the first time in an in vivo setting. The use of this animal model has revealed that the progressive intracellular neuromelanin build‐up that occurs with aging ultimately triggers PD‐like neuronal dysfunction and neurodegeneration once a specific threshold of neuromelanin accumulation is reached. Therefore, although neuromelanin synthesis per se may be neuroprotective, its long‐term accumulation may have deleterious consequences.

From a pathogenic point of view, these results could explain major features of PD, including (1) the selective vulnerability of specific neuronal groups (ie, neurons containing neuromelanin); (2) the correlation of PD with age (as neuromelanin accumulates progressively with age); (3) the progressive nature of the neurodegenerative process (as not all neurons accumulate neuromelanin at the same rate); (4) the known PD‐linked cellular alterations, such as mitochondrial dysfunction, oxidative stress, autophagy/UPS deficits, inclusion body formation or neuroinflammation (all of which may occur secondarily to intracellular neuromelanin accumulation or extracellular neuromelanin release); (5) the uniqueness of PD to humans (as only humans accumulate such high levels of neuromelanin); or (6) the supposed transmission of LB pathology from host PD patients to grafted embryonic mesencephalic neurons (as LB are only observed within aged grafted neurons containing adult levels of neuromelanin).

In a broader context, these results imply that all humans would potentially develop PD if they were to live long enough to reach the pathogenic threshold of intracellular neuromelanin accumulation. Supporting this concept, although only some individuals actually develop PD (1 to up ~5% of the population older than 60, increasing with age),^82^ 10% to 30% of apparently healthy people older than 60 years exhibit PD‐type LB pathology in their melanized brain stem nuclei (ie, incidental LB disease),^83^ and these subjects display intracellular neuromelanin levels above the pathogenic threshold defined in neuromelanin‐producing hTyr‐overexpressing rodents.^43^ In addition, parkinsonian signs such as bradykinesia, stooped posture, and gait disturbance are common in elderly individuals, in the absence of overt PD, reaching a prevalence of up to 50% in individuals older than 80 years of age.^84^, ^85^ Also, multiple reports have documented an age‐related loss of pigmented nigral neurons, estimated at about 10% per decade, in otherwise healthy individuals.^17^ Consistent with age‐dependent alterations of neuromelanin‐containing neurons, brains from aged individuals commonly exhibit a downregulation of dopaminergic phenotypic markers and α‐synuclein accumulations within neuromelanin‐laden SNpc neurons^54^, ^86^ as well as abundant extracellular neuromelanin associated with sustained microglial activation^17^ when compared with young adult brains. Also, in a recent retrospective study on healthy individuals of different ages (from 5‐83 years), the neuromelanin‐sensitive MRI SNpc signal peaked in middle age before declining in older ages, which was attributed to an age‐related decrease of melanized nigral neurons.^87^ Overall, neuromelanin‐filled neurons from apparently healthy aged individuals exhibit early signs of neuronal dysfunction/degeneration. Whether these changes precede clinical PD or are part of normal brain aging remains to be determined. In any case, based on the results discussed here, PD will appear in those subjects that have reached earlier the pathogenic threshold of intracellular neuromelanin accumulation.

From a therapeutic point of view, these results also imply that strategies to maintain or decrease intracellular neuromelanin to levels below the pathogenic threshold should be able to prevent, halt, or delay neuronal dysfunction and degeneration linked to both PD and brain aging (Fig. 2). Because of the putative protective role of neuromelanin synthesis itself (ie, to prevent the cytosolic accumulation of dopamine‐derived toxic species), potential therapies aimed at maintaining or reducing intracellular neuromelanin to levels below the pathogenic threshold should probably favor the elimination of neuromelanin from the cell once neuromelanin has already been produced instead of inhibiting neuromelanin synthesis per se, which may instead prove detrimental. It would be thus important for any therapeutic strategy aimed at modulating neuromelanin levels to tightly regulate neuromelanin levels above its protective threshold and below its pathogenic threshold.

If PD were indeed triggered by an excessive accumulation of intracellular neuromelanin, above a certain pathogenic threshold, then neuromelanin levels might provide an indication of the risk of developing PD. For instance, neuromelanin's binding of iron provides a paramagnetic source for its detection in living brain tissue by MRI.^88^ Currently, neuromelanin‐sensitive MRI is mainly used to differentiate between PD patients and age‐matched healthy controls based on the loss of neuromelanin‐sensitive MRI signal in PD subjects secondary to SNpc neurodegeneration.^88^ However, one could envisage the use of this technique in subjects at risk for PD (eg, nonmanifesting carriers of PD‐linked genetic mutations or subjects with idiopathic rapid eye movement sleep behavior disorder) or even in the general aging population. This would allow to assess whether these subjects might be approaching or reaching the pathogenic threshold of neuromelanin accumulation and thus allow the prompt application of potential neuromelanin modulatory therapies in these subjects to prevent disease. This could also be envisaged with the use of [^18^F]‐AV‐1451 (Flortaucipir), a novel Positron Emission Tomography (PET) tracer that was initially developed for its high affinity to neurofibrillary tau in Alzheimer's disease but was subsequently found to bind strongly and specifically to neuromelanin and melanin.^89^, ^90^ Alternatively, it could be also interesting to determine whether brain neuromelanin levels might correlate with, and thus be inferred from, the production and/or type of pigmentation in the skin or hair. Supporting this concept: (1) PD patients have an increased risk of developing cutaneous melanoma and, reciprocally, patients with cutaneous melanoma have an increased risk of developing PD^91^; (2) similar to neurons, cutaneous melanocytes derive from pluripotent neural crest cells and their maturation is influenced by the same signaling molecules that play a role in central and peripheral nervous tissue^92^; (3) loss‐of‐function variants of the melanocortin 1 receptor gene, which are associated with red hair and fair skin, are linked to an increased risk of both cutaneous melanoma and PD^93^, ^94^; (4) genetically modified mice carrying an inactivating mutation of melanocortin 1 receptor mimicking the human redhead phenotype have compromised nigrostriatal dopaminergic neuronal integrity and are more susceptible to dopaminergic parkinsonian neurotoxins.^95^

## Concluding Remarks and Future Directions

The development of the first rodent model producing human‐like neuromelanin in PD‐vulnerable dopaminergic nigral neurons has allowed for the first time to experimentally assess the consequences of age‐dependent neuromelanin accumulation up to levels reached in elderly humans in an in vivo setting. The observation in this animal model that age‐dependent intracellular neuromelanin build‐up ultimately leads to PD‐like neuronal dysfunction and neurodegeneration when reaching a certain pathogenic threshold of accumulation may have far‐reaching implications for both PD and brain aging.

Among the outstanding questions that remain to be answered is whether neuromelanin synthesis in humans occurs enzymatically, nonenzymatically, or by a combination of both. Although the pathogenic effects linked to neuromelanin accumulation discussed here appear to be independent of the actual mechanisms of neuromelanin synthesis, as they occur downstream of neuromelanin production and subsequent accumulation, the elucidation of the mechanisms of neuromelanin synthesis could provide important clues about the significance of neuromelanin production or identify potential molecular targets to modulate neuromelanin levels. Understanding these mechanisms may also help unravel why PD patients would reach the pathogenic threshold of neuromelanin accumulation earlier than healthy subjects. This could be due, for instance, to an increased or accelerated production of neuromelanin in PD patients caused by an upregulation of hTyr or other related enzymes, such as tyrosinase‐related protein‐1 and tyrosinase‐related protein‐2. Consistent with this, the expression of tyrosinase‐related protein‐2 (ie, dopachrome tautomerase) is increased in dopaminergic neurons derived from induced pluripotent stem cells from PD patients.^96^ Alternatively, increased neuromelanin production in PD could arise from increased levels of free cytosolic dopamine that oxidizes into neuromelanin, for instance, by defects in vesicular monoamine transporter 2–mediated encapsulation of dopamine within synaptic vesicles, as observed in early PD cases.^97^ Indeed, there is an inverse relationship between neuromelanin content and vesicular monoamine transporter 2 immunoreactivity in human midbrain dopaminergic neurons, with neurons exhibiting the highest vesicular monoamine transporter 2 levels and the lowest neuromelanin levels being the less vulnerable to PD‐linked neurodegeneration.^63^

It would also be important to further uncover the exact molecular mechanisms driving neuromelanin‐linked pathology. In addition of the potential detrimental effects of a physical cytosolic overcrowding by neuromelanin or the formation of potentially toxic dopamine‐derived oxidized species during neuromelanin synthesis, other factors could also be considered. For instance, neuromelanin has the ability to chelate various transition metal ions^98^ and neuromelanin is in fact the main iron storage molecule in SNpc dopaminergic neurons.^99^, ^100^ The equilibrium between iron, dopamine, and neuromelanin seems to be crucial for cell homeostasis,^99^, ^101^ and PD brains exhibit increased levels of iron when compared with healthy control subjects.^100^ In this context, neuromelanin may represent a potential source of toxic iron if the iron‐binding capacity of this pigment becomes saturated.^101^ Similarly, the capacity of neuromelanin to bind environmental parkinsonian neurotoxins, such as 1‐methyl‐4‐phenylpyridinium (1‐methyl‐4‐phenyl‐1,2,3,6‐tetrahydropyridine MPTP's active metabolite) or the pesticide paraquat could also be considered.^102^ For instance, it has been observed in macaques that among dopaminergic neurons, those containing neuromelanin are more susceptible to MPTP toxicity than nonmelanized neurons.^103^

These and other questions could now be addressed in vivo for the first time by means of the newly available neuromelanin‐producing rodent model.^43^ This model could be used for (1) the study of the synthesis, biology, and pathogenic mechanisms of neuromelanin in vivo in both aging and disease, (2) the screening of novel therapeutic strategies aimed at modulating neuromelanin levels within the aged or diseased brain, and (3) the search for potential neuromelanin‐related biomarkers for the risk, early detection, diagnosis, and/or progression of PD. The introduction into experimental in vivo research of a factor such as neuromelanin that is so intimately linked to PD and that has thus far been neglected in PD animal modeling should open new research avenues and could lead to a paradigm shift in the field of PD and, in a broader sense, brain aging.

## Full financial disclosure for the previous 12 months
